# Macrophage-derived exosomal miR-342-3p promotes the progression of renal cell carcinoma through the NEDD4L/CEP55 axis

**DOI:** 10.32604/or.2022.03554

**Published:** 2022-10-10

**Authors:** JIAFU FENG, BEI XU, CHUNMEI DAI, YAODONG WANG, GANG XIE, WENYU YANG, BIN ZHANG, XIAOHAN LI, JUN WANG

**Affiliations:** 1 NHC Key Laboratory of Nuclear Technology Medical Transformation (Mianyang Central Hospital), Mianyang, 621000, China; 2 Departments of Clinical Laboratory, Mianyang Central Hospital, School of Medicine, University of Electronic Science and Technology of China, Mianyang, 621000, China; 3 Medical Technology Institute, Chengdu University of Traditional Chinese Medicine, Chengdu, 611137, China; 4 Departments of Urology Surgery, Mianyang Central Hospital, School of Medicine, University of Electronic Science and Technology of China, Mianyang, 621000, China; 5 Departments of Pathology, Mianyang Central Hospital, School of Medicine, University of Electronic Science and Technology of China, Mianyang, 621000, China; 6 Department of Medical Laboratory, Affiliated Hospital of Southwest Medical University, Luzhou, 646000, China

**Keywords:** Renal cell carcinoma, M2 macrophage, miR-342-3p, NEDD4L, CEP55, PI3K/AKT/mTOR signaling pathway

## Abstract

Due to its difficulty in early diagnosis and lack of sensitivity to chemotherapy and radiotherapy, renal cell carcinoma (RCC) remains to be a frequent cause of cancer-related death. Here, we probed into new targets for its early diagnosis and treatment for RCC. microRNA (miRNA) data of M2-EVs and RCC were searched on the Gene Expression Omnibus database, followed by the prediction of the potential downstream target. Expression of target genes was measured via RT-qPCR and Western blot, respectively. M2 macrophage was obtained via flow cytometry with M2-EVs extracted. The binding ability of miR-342-3p to NEDD4L and to CEP55 ubiquitination was studied with their roles in the physical abilities of RCC cells assayed. Subcutaneous tumor-bearing mouse models and lung metastasis models were prepared to observe *in vivo* role of target genes. M2-EVs induced RCC growth and metastasis. miR-342-3p showed high expression in both M2-EVs and RCC cells. M2-EVs carrying miR-342-3p promoted RCC cell abilities to proliferate, invade and migrate. In RCC cells, M2-EV-derived miR-342-3p could specifically bind to NEDD4L and consequently elevate CEP55 protein expression via suppressing NEDD4L, thereby exerting tumor-promoting effects. CEP55 could be degraded by ubiquitination under the function of NEDD4L, and miR-342-3p delivered by M2-EVs facilitated the RCC occurrence and development by activating the PI3K/AKT/mTOR signaling pathway. In conclusion, M2-EVs promote RCC growth and metastasis by delivering miR-342-3p to suppress NEDD4L and subsequently inhibit CEP55 ubiquitination and degradation via activation of the PI3K/AKT/mTOR signaling pathway, strongly driving the proliferative, migratory and invasive of RCC cells.

## Introduction

Renal cell carcinoma (RCC) represents the most frequent type of renal neoplasm, accounting for 90% of renal malignancies and 2%~3% of all adult malignant cancers [1,2]. It is the most lethal neoplasm of the urologic system, defined by an asymptomatic disease course, with late and uncharacteristic symptoms, leading to a poor survival prognosis [3]. Since the 1990s, the incidence of RCC has steadily increased year by year, with an annual increase of approximately 2% to 3% [2,4]. Patients suffering from localized RCC are frequently treated with nephrectomy [5]. Unfortunately, metastasis occurs in approximate one-quarter of the patients, causing meager survival rates and severe social burden [6,7]. Therefore, identification of novel biomarkers is crucial for the treatment of RCC.

Macrophages are the most abundant immune cells in the tumor microenvironment (TME) and can be categorized into the classically activated (M1) and alternatively activated (M2) macrophages according to the polarization status [8,9]. M1 macrophages exhibit tumoricidal activities, whereas M2 macrophages facilitate tumor progression by enhancing tumor angiogenesis and metastasis [9,10]. Numerous types of cells, including macrophages, release extracellular vesicles (EVs), which are bioactive membrane-enclosed “packages” containing proteins, lipids and nucleic acids that are capable of modulating the TME and affecting the signaling pathway of recipient cells [11,12]. Increasing evidence suggests that cancers hijack EV-mediated communication to facilitate tumor progression by promoting cell proliferation, sustaining angiogenesis, reprogramming energy metabolism, evading immune response, and contributing to cancer cell invasion and metastasis [12–14]. In particular, M2 macrophage-derived EVs have been shown to stimulate the migration and invasion of various cancer types, including colorectal cancer, hepatocellular cancer and pancreatic cancer [15–17]. MicroRNAs (miRNAs) are one of the most studied classes of biomolecules carried by EVs. miRNAs, small non-coding RNAs, are capable of regulating gene expression post-transcriptionally, resulting in the attenuated translation of target mRNAs [18]. Several EV-derived miRNAs such as miR-19b-3p, miR-30c-5p and miR-210 have been studied and proposed as potential diagnostic and therapeutic targets in clear cell RCC (ccRCC, the most common subtype of RCC accounting for 75%–80% of all RCC cases) [19–21]. However, due to the novelty of this field, the role of M2 macrophage-derived EVs (M2-EVs) in regulating RCC progression and metastasis still needs to be further elucidated.

Herein, we first determined M2-EVs-related miRNAs by bioinformatics analysis in RCC and identified miR-342-3p as the significantly up-regulated miRNAs. The role of miR-342-3p in several cancers (such as hepatocellular and nasopharyngeal carcinoma) has already been investigated [22,23]. However, how EV communication involving miR-342-3p affects RCC cell is not fully understood. In this study, we explored the role of M2-EV-derived miR-342-3p in RCC and discussed the underlying mechanisms.

## Materials and Methods

### Bioinformatics analysis

M2 macrophage-derived EV dataset GSE97467 and RCC datasets GSE71302, GSE95384 and GSE36895 were searched from the Gene Expression Omnibus database. GSE97467 contains EV data derived from THP-1 monocyte (n = 4) and M2 macrophage (n = 4), while GSE71302 (normal, n = 5; tumor, n = 5), GSE95384 (normal, n = 8; tumor, n = 8) and GSE36895 (normal, n = 23; tumor, n = 29) includes RCC and normal renal tissue samples. Differentially expressed miRNAs that met |logFC| > 1 and *P* value < 0.05 were screened out using the R package “limma” package. ENCORI database was used to obtain potential targets for miR-342-3p and perform correlation analysis. Additionally, E3 ubiquitin ligases were predicted through the Ubibrowser database. Starbase was searched to obtain binding sites of miR-342-3p and NEDD4L.

### Cell culture

RCC cell lines (ACHN and 769-P), human umbilical vein endothelial cells (HUVECs) and monocyte cell line THP-1 were purchased from the Cell Bank of the Typical Culture Preservation Committee of Chinese Academy of Sciences (Shanghai, China). These cell lines were cultured in RPMI 1640 medium supplemented with 10% fetal bovine serum (FBS) at 37°C in a 5%CO_2_ humidified incubator.

### Isolation of CD11b+/CD163+ M2 TAMs

RCC tissue samples were cut into small pieces, digested at 37°C and subjected to the continuous rotation to remove erythrocytes. Then, cells were incubated with Alexa Fluor 488-conjugated mouse anti-human CD11b (R&D Systems, Minneapolis, MN, USA, FAB16991G-100) and mouse anti-human CD163-APC (Miltenyi, Shanghai, China, 130-100-612) or control IgG antibodies (Miltenyi, Shanghai, China, 130-098-846 and R&D Systems, Minneapolis, MN, USA, IC0041G) at 4°C for 30 min. CD11b^+^/CD163^+^ M2 tumor-associated macrophages (TAMs) were sorted out via a flow cytometer (BD FACSAria II cell sorter).

### Immunofluorescent staining

M2 TAMs were digested and cultured in an immunofluorescence chamber (2 × 10^5^ cells/per well). When the cell confluence reached about 90%, the cells were washed with cold PBS three times and fixed in 4% paraformaldehyde. After permeabilizing with 0.3% Triton X-100 and blocking with goat serum, the cells were incubated with the primary antibodies (diluted in PBS at a ratio of 1:100, Abcam, Cambridge, UK) CD68 (ab213363), CD206 (ab125028) and CD163 (ab156769) at 4°C overnight. Afterward, the cells were incubated with FITC-labeled fluorescent secondary antibody (sheep anti-mouse IgG H&L) (Abcam, Cambridge, UK) at room temperature away from light for 1 h. The cells were then exposed to 4′,6-diamidino-2-phenylindole (DAPI) (100 μL per well) for 15 min in dark conditions, sealed and observed under a fluorescence microscope (ECLIPSE E800, Nikon, Tokyo, Japan).

### Lentiviral infection and plasmid transfection

For lentiviral infection, available M2 TAMs were trypsin-digested, counted and then inoculated in 6-well plates. Lentivirus vectors containing miR-342-3p inhibitor (anti-miR-342-3p) and corresponding negative control (anti NC) (Genechem Inc., Shanghai, China) (MOI = 20) were transfected into M2 TAMs. Highly transfected cells were sorted out using a 3-day treatment of puromycin (1 μg/mL). M2 TAMs were divided into the following groups: control (normal saline), M2-CM (conditioned medium [CM] for extraction of M2 TAMs), M2-anti-NC and M2-anti miR-342-3p. TAMs of these groups were infected respectively with lentivirus for EV isolation.

Transient transfection was fulfilled in RCC cells with pcDNA-3.1 NEDD4L, pcDNA-3.1 Smurf1, pcDNA-3.1 Smurf2, pcDNA-3.1 Itch, CEP55 shRNA, miR-342-3p mimic, miR-342-3p inhibitor and corresponding NCs (GenechemInc., Shanghai, China) respectively. In short, RCC cells at 4 × 10^5^ cells/mL were counted and cultured in 6-well plates. Upon reaching 80% confluence, cell transfection was performed using Lipofectamine 2000.

### Isolation and identification of M2-EVs

M2 TAMs were incubated overnight at 2 × 10^5^ cells per well in 6-well plates. The culture medium was replaced with EV-free serum. After being cultured for another 48 h, CM was collected, and M2-EVs were isolated via differential centrifugation (500 g for 15 min; 2,000 g for 15 min; 10,000 g for 20 min) at 4°C. Following filtration by a 0.22 µM filter (on ice), the sample was centrifuged at 110,000 g for 70 min, resuspended using PBS (on ice) and ultracentrifuged. M2-EVs were finally obtained after resuspension in 100 μL sterile PBS.

A transmission electron microscope (TEM) was adopted to validate the successful extraction of M2-EVs. Briefly, 20 µL EVs were loaded onto the copper grid, followed by liquid suction with filter paper, counterstaining with the addition of 30 µL phosphotungstic acid solution (pH 6.8), baking with incandescent light and photographing. Particle size was determined by a Nanoparticle Tracking Analyzer (NS300, Malvern Instruments, Ltd., UK).

Surface markers of EVs were quantitated by Western blot. In short, the concentrated EV suspension was firstly processed by a bicinchoninic acid (BCA) kit (23227, Thermo Fisher Scientific, Waltham, MA, USA) for protein quantitation. Then, the proteins were separated by SDS-PAGE and transferred to a bio-membrane to determine the expression of EV markers, including Alix (ab117600, 1:1000), CD63 (ab134045, 1:1000), LAMP2 (ab13524, 1:1000) and calnexin (ab22595, 1:1000). All antibodies were procured from Abcam. Ponceau red was used as a loading control.

### Fluorescent labeling and transfer of EVs

M2-EV labeling was completed with the PKH67 Green Fluorescence Kit (UR52303, Umibio, Shanghai, Co., Ltd., China). Afterward, the harvested EVs were co-cultured with RCC cells, fixed in 4% paraformaldehyde, washed by PBS and exposed to DAPI (Sigma, MO, USA, D9542) for nuclear staining. The slides were photographed under the fluorescence microscope (ECLIPSE E800, Nikon, Tokyo, Japan).

The internalization of M2-EV-carried miR-342-3p by recipient cells (RCC cells) was further investigated. M2 macrophages were transfected with Cy3-miR-342-3p (GenePharma) using Lipofectamine 3000 (Invitrogen, Carlsbad, CA, USA) (serum-free medium) for 6 h, and then the medium was replaced with 10% EV-free serum medium. After further 48-h incubation, the supernatant was collected, and M2-EVs carrying Cy3-miR-342-3p were extracted. The EVs were resuspended by PBS and added to RCC cells. Subsequently, the cells were fixed and washed by PBS, and the cytoskeletons were labeled with Phalloidin-iFfluor 488 Reagent (green, ab176753, 1:1000, Abcam, Cambridge, UK) for 30 min at room temperature. The nuclei were stained with DAPI. Finally, the internalization of EVs-miR-342-3p in RCC cells was validated microscopically by fluorescence microscopy (ECLIPSE E800, Nikon, Japan).

### Dual-luciferase reporter assay

A dual luciferase reporter assay was done using a Promega dual-luciferase reporter kit (Madison, Wisconsin, USA). The sequences of NEDD4L containing predicted miR-342-3p binding sites and mutant binding sites were ligated to the pmirGLO Dual-Luciferase miRNA Target Expression Vectors (E1330, Promega, Madison, Wisconsin, USA) to build NEDD4L-WT and NEDD4L-MUT reporter vectors. HEK293 cells were co-transfected with the above-indicated plasmids and miR-342-3p mimic or miR-NC using Lipofectamine 3000 (Invitrogen, Carlsbad, CA, USA). After 48 h, the luciferase activities were detected.

### Co-immunoprecipitation (Co-IP)

RCC cells were collected and lysed with RIPA (C1053, Beijing Pulley Gene Technology Co., Ltd., Beijing, China) containing protease inhibitor cocktail (HY-K0010, MCE, Shanghai, China). The obtained proteins were incubated with primary antibodies (anti-Flag, AE005, 1:100, mouse antibody; anti-Myc, AE070, 1:50, rabbit antibody, Abclonal, Woburn, MA, USA) at 4°C overnight. Protein A/G-agarose beads (36403ES08, Yeasen, Shanghai, China) were added for 4 h to obtain immunoprecipitates, which were then purified at least three times in a lysis buffer. Purified complexes were treated by 2 × SDS loading buffer, boiled, electrophoresed and assayed by Western blot.

### GST pull-down assay

*E. coli* (BL21 DE3 strain) transformed with GST-NEDD4L or His-CPE55 vectors was induced with 0.4 mM IPTG at 16°C for 16 h to express GST-NEDD4L or His-CPE55 proteins. Purified GST-NEDD4L proteins (200 ng) were immobilized on glutathione-agarose beads (G0924-1ML, Millipore, Burlington, MA, USA), which were then incubated with purified HIS-CPE55 proteins (200 ng). GST protein alone served as a negative control. After washing, a Western blot assay was performed to analyze the proteins bound to the beads using anti-His (catalog sc-803, Santa Cruz Biotechnology Inc., Santa Cruz, CA, USA, 1:1000) or anti-GST antibody (catalog sc-138, Santa Cruz Biotechnology Inc., Santa Cruz, CA, USA, 1:5000).

### Endogenous ubiquitination analysis

HA-Ub, Myc-NEDD4L, Myc-NEDD4L-MUT or Flag-CEP55 plasmids were transfected into RCC cells, followed by 4-h treatment with 10 μM MG132 (MedChemExpress, Shanghai, China). Afterward, cell lysis was performed using 100 µL conventional lysis buffer. The products were denatured (95°C, 5 min), added with 1% SDS and incubated in anti-Flag antibody (AE005, 1:100, mouse antibody, Abclonal, Woburn, MA, USA) and protein G-agarose (11243233001, Roche, Shanghai, China) successively. Anti-ubiquitin antibody was used to determine the endogenous ubiquitination of CEP55 in immunoprecipitates by Western blot.

### Protein half-life assay

RCC cells were treated with 10 μM cycloheximide (CHX, MedChemExpress, Shanghai, China) for various periods (0, 0.5, 1 and 2 h), or treated with MG132 to block protein synthesis at the same time. Cell extracts were prepared for Western blot assay.

### CCK-8 assay

Cells were cultured in the 96-well culture plates (100 μL, 3 × 10^4^/mL). Accordingly, 10 μL CCK-8 (Sigma, Saint Louis, MO, USA) was added to each well. After being incubated in the cell incubator for 1 h, the absorbance at 450 nm was measured on the enzyme labeling instrument (NYW-96M, Beijing Nuoyawei Instrument Co., Ltd., Beijing, China). The cell viability curve was drawn with time as abscissa and optical density value as ordinate.

### Transwell assay

Cells were added to the upper Transwell chambers (24-well insert; pore size, 8 μm; Corning, Tewksbury, MA, USA) pre-coated without (migration assay) or with (invasion assay) 50 μL of diluted Matrigel (1:8 dilution; BD Biosciences). Medium containing 10% FBS was added to the lower Transwell chambers. After 48 h, the transmigrated cells were fixed with 4% paraformaldehyde, treated with 0.2% Triton X-100 (Sigma, Saint Louis, MO, USA) solution and stained with 0.05% gentian purple. The number of stained cells was counted under an inverted microscope (XDS-800D, Shanghai Caikang Optical Instrument Co., Ltd., Shanghai, China) to evaluate the cell migration and invasion capabilities. Five visual fields were randomly selected to count, and the number of cells was expressed by mean.

### RT-qPCR

Total RNA extracted from tissues or cells using Trizol (Thermo Fisher Scientific, Waltham, MA, USA) was taken to perform reverse transcription to obtain cDNA or miRNA cDNA with a reverse transcription kit (Fermentas Inc., Ontario, CA, USA) or miRcute Plus miRNA First-Strand cDNA Synthesis Kit (TIANGEN, China). Synthetic exogenous internal reference cel-miR-39 (1 pmol per sample; TIANGEN, Beijing, China) was supplemented to the culture medium (350 μL) or EVs (100 μg) before the experiment. Subsequently, miRNA was isolated from these samples using the mirVana PARIS kit (Ambion, Austin, Texas, USA). RT-qPCR for mRNA was completed with SYBR® Premix Ex Taq (Takara, Tokyo, Japan) on the ABI StepOne real-time PCR system (Applied Biosystems, Dublin, CA, USA). Meanwhile, miRcute Plus miRNA qPCR detection kit (TIANGEN, Beijing, China) was adopted for RT-qPCR of miRNA. GAPDH was used as the internal control for measuring mRNA expression in cells and tissues, while U6 for measuring miRNA expression. In addition, the miRNA level of culture medium and EVs was normalized to exogenous cel*-*miR*-*39. The universal reverse primer of miRNA was obtained from miRcute Plus miRNA qPCR detection kit (TIANGEN, Beijing, China), and other primers were provided by Shanghai Sangon (Shanghai, China). All primer sequences were displayed in Table 1. All experiments were processed in triplicates, and the results were analyzed following the 2^(*−*ΔΔCT)^ method.

**Table 1:** RT-qPCR primer sequences

Gene	Primer sequences (5′-3′)
miR-342-3p (human)	F: TCTCACACAGAAATCGC
	R: universal primer
cel-miR-39 (human)	F: GGTCACCGGGTGTAAATCAGCTTG
	R: universal primer
U6 snRNA (human)	F: CTCGCTTCGGCAGCACA
	R: universal primer
NEDD4L (human)	F: GACATGGAGCATGGATGGGAA
	R: GTTCGGCCTAAATTGTCCACT
GAPDH (human)	F: CATCTTCTTTTGCGTCGCCA
	R: TTAAAAGCAGCCCTGGTGACC

### Western blot

Total protein was extracted from tissues or cells using RIPA lysate containing PMSF (Applygen Gene Technology Corp., Beijing, China). Protein concentration was determined based on the instructions of a BCA protein assay kit (Thermo Fisher Scientific, Waltham, MA, USA). The protein bands were separated and transferred to a polyvinylidene fluoride membrane (Millipore, Burlington, MA, USA). The membrane was blocked with 5% skim milk for 1 h. Primary antibodies (all from Abcam, Cambridge, UK) against NEDD4L (ab46521, 1:1000), CEP55 (ab170414, 1:1000), PI3K (ab32089, 1:500), AKT (ab8805, 1:1000), p-AKT (ab38449, 1:1000), mTOR (ab32028, 1:1000), p-mTOR (ab109268, 1:1000), E-cadherin (ab40772, 1:500), Vimentin (ab92547, 1:1000) and GAPDH (ab8245, 1:500) were added for incubation at 4°C overnight. Then, the membranes were incubated with HRP-coupled goat anti-rabbit IgG second antibody (ab150077, 1:1000, Abcam, Cambridge, UK) for 1 h at room temperature. Subsequently, an enhanced chemiluminescence solution (Biomiga, San Diego, CA, USA) was added for color development. A Bio-Rad Gel Doc EZ Imager (Bio-Rad, Hercules, CA, USA) was applied for image capture, and Image Pro Plus 6.0 software (Media Cybernetics, Rockville, MD, USA) was used for quantitation. The density of each protein band was normalized to GAPDH. Semi-quantitative analysis was performed based on the ratio of the mean gray value of the target protein to the mean gray value of internal reference.

### Xenograft tumor and lung metastasis in nude mice

Sixty-four 4-5-week immunodeficient nude mice (Bal B/c, nu/nu) were housed under non-pathogenic conditions at 20°C–26°C and humidity 50%–65%. ACHN cells or ACHN cell lines stalely transfected with pcDNA-3.1-NEDD4L were collected, counted, and resuspended in PBS with the final concentration of 2 × 10^7^ cells/mL. In addition, trypan blue exclusion determined that 95% of the cells were viable prior to injection. The prepared ACHN cells were subcutaneously injected into the back of nude mice. Eight days after injection, DiI-labeled EVs or PBS (blank control) were injected through the tail vein. After that, the tumor volume was measured at six-day intervals with a vernier caliper and calculated as W = 1/2 * a * b^2^ (a, long diameter; b, short diameter). Four weeks later, CO_2_ was used to execute nude mice, and tumor tissues was isolated and weighed. The nude mice were randomly assigned to: i. Control (tail vein injection of saline); ii. M2-EVs (tail vein injection of 10 μg M2-EVs); iii. M2-EVs-anti miR-342-3p (tail vein injection of 10 μg EVs isolated from M2 macrophages infected with 10 μg anti-miR-342-3p lentivirus); iv. Overexpressed (oe)-NEDD4L (injection of stable pcDNA-3.1-NEDD4L transduced ACHN cell line and tail vein injection of saline); v. oe-NEDD4L+M2-EVs (injection of stable pcDNA-3.1-NEDD4L transduced ACHN cell line and tail vein injection of M2-EVs) (n = 8 for each treatment).

To establish lung metastasis model, ACHN cells or ACHN cells stably transfected with pcDNA-3.1-NEDD4L were injected into female Bal B/C nude mice through the tail vein (1 × 10^6^ cells/100 μL). After 14 days, 10 μg DiI-labeled EVs or an equal amount of PBS was repeatedly injected into nude mice via a caudal vein twice a week for one month. Subsequently, all nude mice were euthanized. Then, metastatic pulmonary nodes were counted, and the lung weight was measured. Lung tissues were harvested to evaluate the expressions of miR-342-3p, NEDD4L and CEP55 via RT-qPCR and Western blot. The lung was split for further H&E staining.

### Statistics

All data were processed by SPSS 21.0 statistical software (SPSS, Inc., Chicago, IL, USA) and GraphPad Prism 8.0.2 software (GraphPad Software, Inc., La Jolla, CA, USA). Measurement data were expressed in the form of mean ± standard deviation. Data of the two groups were compared using the independent samples *t*-test. One-way ANOVA (followed by Tukey posthoc test) was adopted for data comparison between multiple groups. Data were compared between groups at different time points using repeated measures ANOVA and Tukey posthoc test. Values of *p* < 0.05 were considered statistically significant.

## Results

### M2-EVs promote malignant biological behaviors of RCC cells

**Figure 1:**
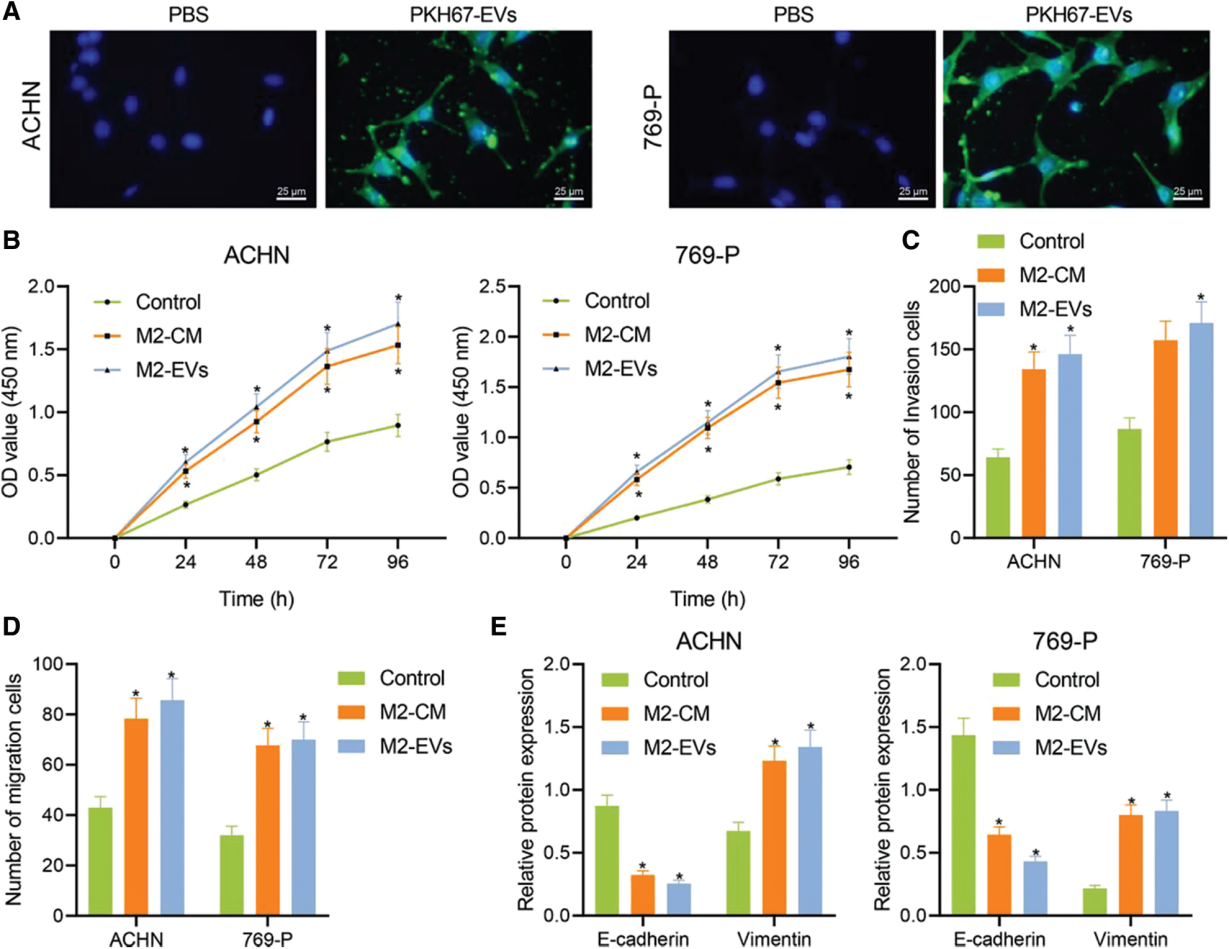
M2-EVs promote proliferation, migration and invasion of RCC cells. (A) Internalization of PKH67-labeled EVs by RCC cells (scale bar = 25 μm); (B) Cellular proliferation activities of RCC cells co-cultured with M2-CM or M2-EVs by CCK-8 assay; (C, D) Migration and invasion abilities of RCC cells co-cultured with M2-CM or M2-EVs by Transwell assay; (E) Western blot analysis for EMT-associated proteins including E-cadherin and Vimentin on RCC cells co-cultured with M2-CM or M2-EVs. * indicates *p* < 0.05 when compared with control group. Measurement data were expressed in the form of mean ± standard deviation (*n* = 3). Data of the two groups were compared using independent samples *t*-test. One-way ANOVA (followed by Tukey posthoc test) was electively used for data comparison between multiple groups. Data were compared between groups at different time points using repeated measures ANOVA and Tukey posthoc test.

**Figure S1:**
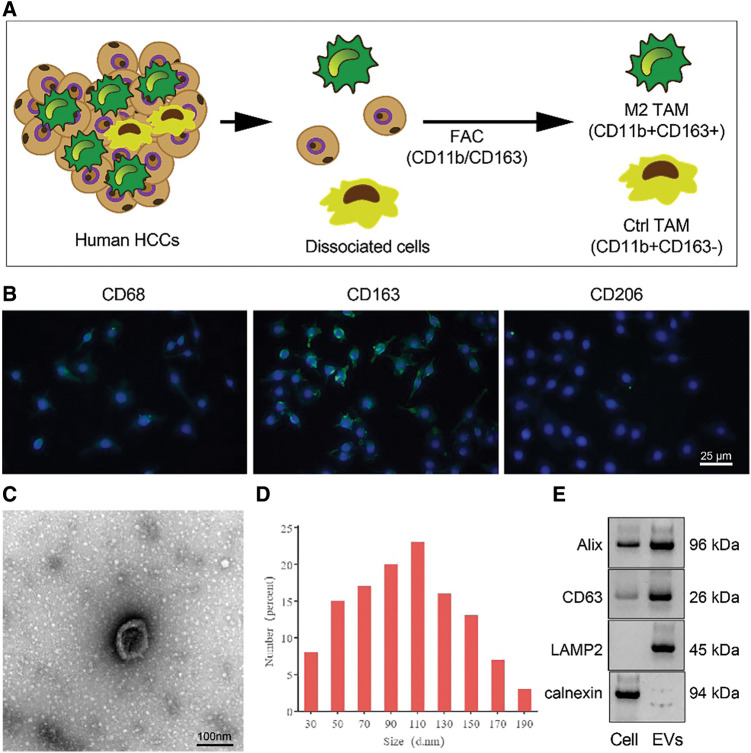
Identification of M2 TAMs from RCC tissues. (A) Isolation and purification of M2 TAMs from RCC tissues; (B) Immunofluorescence staining of M2 TAMs markers CD68, CD163 and CD206 (scale bar = 25 μm); (C) TEM manifestations of M2-EVs (scale bar = 100 nm); (D) Particle size distribution of M2-EVs; (E) Western blot analysis for EVs surface markers including Alix, CD63, LAMP2 and calnexin.

**Figure S2:**
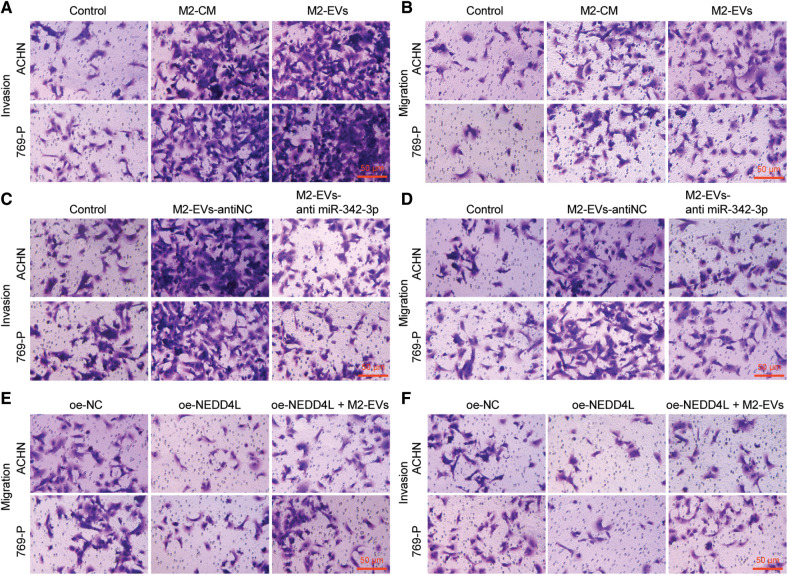
Migration and invasion abilities of RCC cells. (A, B), Representative images of migration and invasion abilities of RCC cells in Fig. 1C and 1D; (C, D), Representative images of migration and invasion abilities of RCC cells in Fig. 3D and 3E; (E, F), Representative images of migration and invasion abilities of RCC cells in Fig. 5C and 5D.

**Figure S3:**
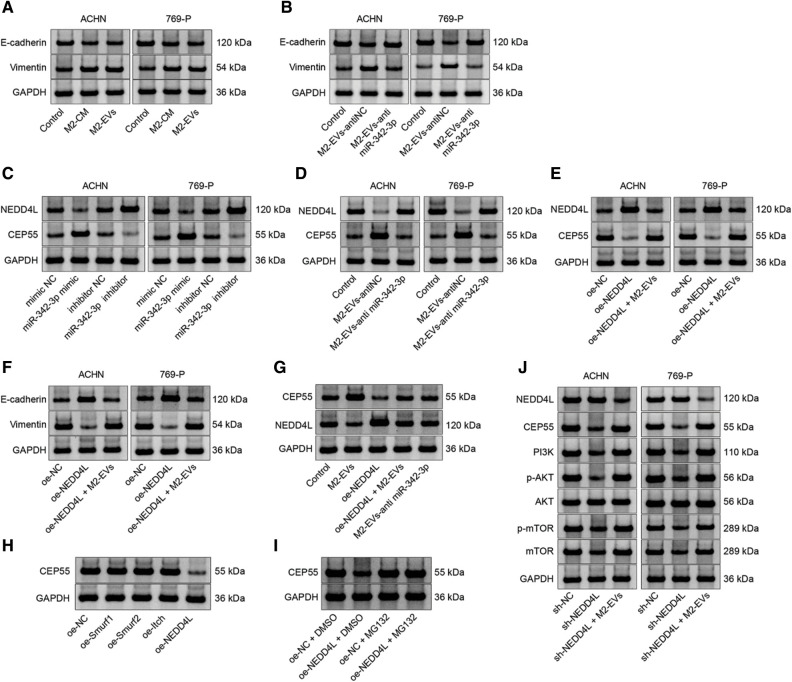
Representative protein bands. (A), Fig. 1E; (B), Fig. 3F; (C), Fig. 4G; (D), Fig. 4H; (E), Fig. 5A; (F), Fig. 5E; (G), Fig. 7F; (H), Fig. 8B; (I), Fig. 8C; (J), Fig. 8G.

M2-EVs bear great responsibility for cell migration and metastasis in several cancers [24]. To investigate the pivotal biological effects of M2-EVs on RCC, M2 TAMs were firstly extracted (Fig. S1A) and presented with positive expressions of markers CD68, CD163 and CD206 in immunofluorescence staining (Fig. S1B). M2-EVs were subsequently extracted. TEM manifested that M2-EVs showed round or oval shapes, which was uneven in size with a complete membrane structure, and contained low-density substances (Fig. S1C). The particle diameter of the EVs was 30–100 nm (Fig. S1D). Western blot assay obtained positive Alix, CD63 and LAMP2 while negative calnexin proteins in EVs (Fig. S1E). PKH67 labeled M2-EVS was co-cultured with RCC cells. PKH67 labeled green fluorescence could be observed in RCC cells (Fig. 1A), indicative of the internalization of M2-EVs by RCC cells. The biological effect of M2-EVs was further analyzed. Compared to the control group, M2-CM and M2-EVs groups showed a significant increase in cell proliferation (Fig. 1B), invasion (Fig. 1C, Fig. S2A) and migration (Fig. 1D, Fig. S2B), accompanied by distinctly down-regulated E-cadherin level and up-regulated Vimentin level (Fig. 1E, Fig. S3A). These results identify that M2-EVs can promote RCC cell abilities to proliferate, migrate and invade.

### M2-EVs derived miR-342-3p facilitates malignant behaviors of RCC cells

**Figure 2:**
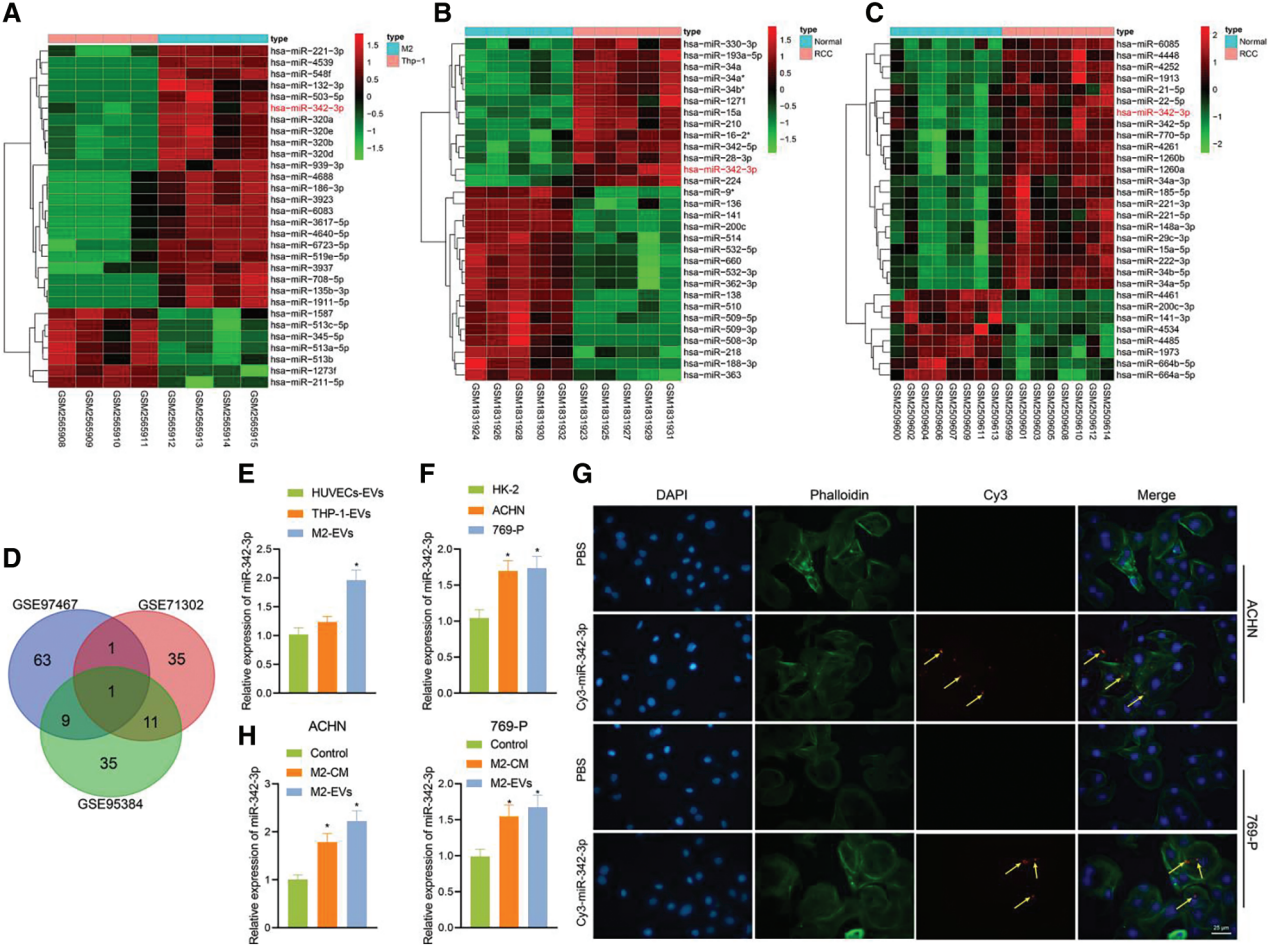
M2-EVs delivers miR-342-3p to RCC cells. (A) Heatmap of the top 30 differentially expressed miRNAs between the THP-1-derived EVs (n = 4) and M2-EVs (n = 4) samples in GSE97467; (B) Heatmap of the top 30 differentially expressed miRNAs between normal kidney (n = 5) and RCC tissue (n = 5) samples in GSE71302; (C) Heatmap of the top 30 differentially expressed miRNAs between normal kidney (n = 8) and RCC tissue (n = 8) samples in GSE95384 database; (D) Venn diagram showing the overlap of up-regulated miRNAs in the three miRNA datasets; (E) RT-qPCR assay for miR-342-3p expression in various EVs obtained from different cells (* indicates *p* < 0.05, compared to the HUVECs-EVs group); (F) Detection of miR-342-3p expression in different cell lines; (G) Internalization of M2-EVs carrying Cy3-miR-342-3p by RCC cells (scale bar = 25 μm) (Red: cy3-labled miR-342-3p, yellow arrow; Green: cytoskeleton by Phalloidin; Blue: nucleus by DAPI); (H) RT-qPCR assay for miR-342-3p expression in RCC cells co-cultured with M2-CM or M2-EVs. * indicates *p* < 0.05, compared with control group. Measurement data were expressed in the form of mean ± standard deviation (*n* = 3). One-way ANOVA (followed by Tukey posthoc test) was electively used for data comparison between multiple groups.

M2-EVs have been shown to facilitate the motility of cancer cells [24]. To know the underlying mechanism of action, miRNA enriched in M2-EVs GSE97467 dataset and differentially up-regulated miRNA data in GSE71302 and GSE95384 dataset were selected and intersected with miR-342-3p identified, which displayed high expression in both M2-EVs and RCC samples (Figs. 2A–2D). Expression of miR-342-3p in M2-EVs, HUVEC-EVs and THP-1-derived EVs was measured via RT-qPCR. The results showed a significant difference in the expression of miR-342-3p in the observed cell-derived EVs (F = 21.302, *p* < 0.001). Compared with HUVEC-EVs and THP-1-derived EVs, miR-342-3p expressed highest in M2-EVs, but its increase was not statistically different from THP-1-EVs (q = 2.169, *p* = 0.463) (Fig. 2E). Additionally, determination of miR-342-3p expression in HK-2, ACHN and 769-P cells detected by RT-qPCR revealed that compared with HK-2, miR-342-3p expression was significantly higher in ACHN and 769-P cells (Fig. 2F). RCC cells were co-cultured with TAMs containing Cy3-miR-342-3p. Microscopically red fluorescence was visible in RCC cells (Fig. 2G). Besides, the miR-342-3p level was found to elevate in both M2-CM (ACHN: q = 7.874, *p* = 0.003; 769-P: q = 6.641, *p* = 0.008) and M2-EVs (ACHN: q = 12.326, *p* < 0.001; 769-P: q = 8.169, *p* = 0.003) groups when compared with the control group, and the elevated level in M2-EVs group was higher than that in M2-CM group (all *p* < 0.05) (Fig. 2H). These results suggest that M2 TAMs can deliver miR-342-3p to RCC cells via EVs.

**Figure 3:**
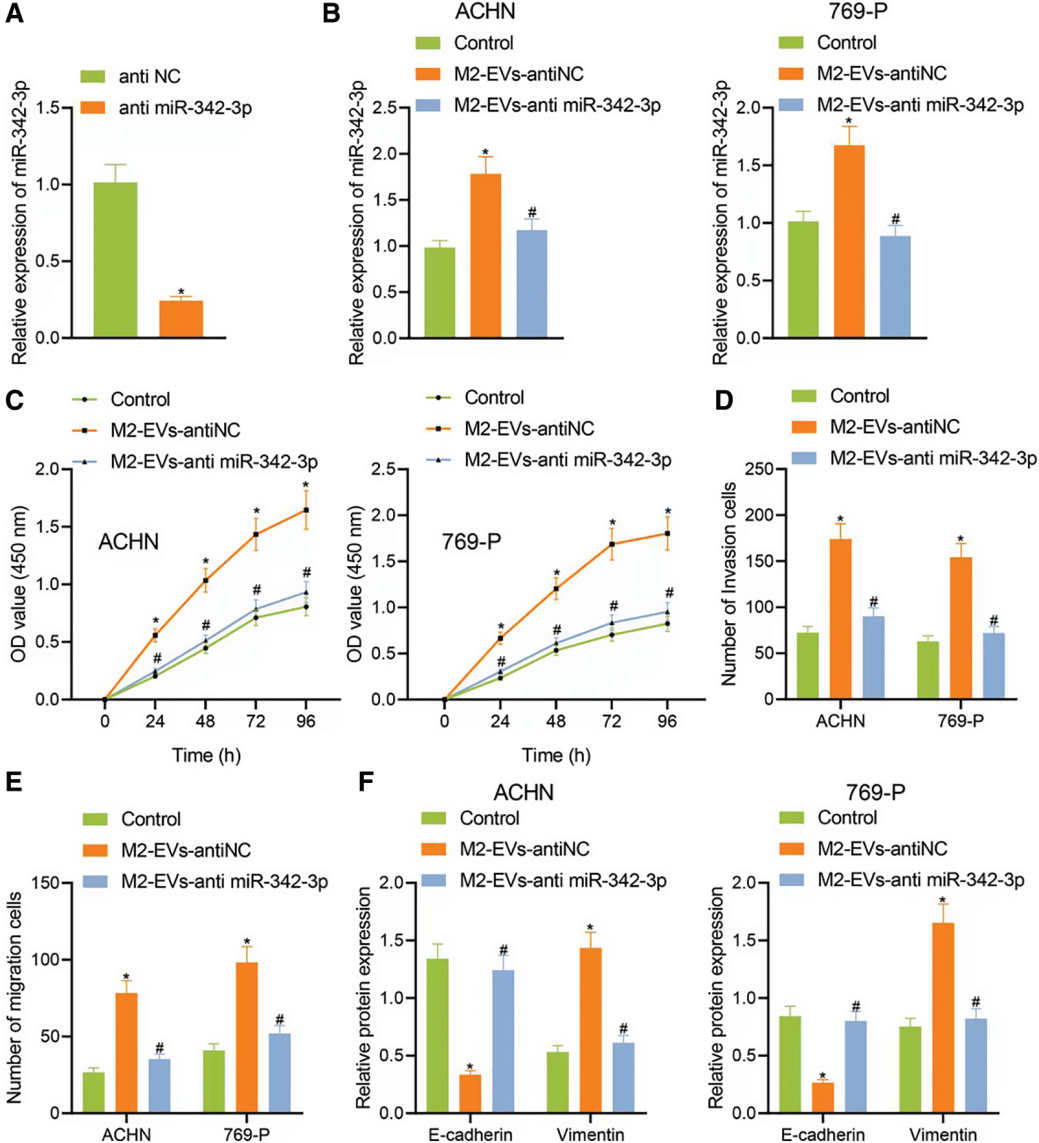
M2-EVs-miR-342-3p promotes proliferation, invasion and migration of RCC cells. (A) RT-qPCR assay for miR-342-3p expression in M2-EVs after transfection of anti-NC or anti-miR-342-3p on M2 TAMs; (B) RT-qPCR assay for miR-342-3p expression in RCC cells co-cultured with M2-EVs-anti-NC or M2-EVs-anti-miR-342-3p; (C–E) Abilities of proliferation, migration and invasion on RCC cells in each treatment group by CCK-8 and Transwell assays; (F) Expression of E-cadherin and Vimentin proteins on RCC cells in each treatment group by Western blot. * indicates *p* < 0.05, compared to anti NC or Control, # represents *p* < 0.05, compared to M2-EVs-anti NC. Measurement data were expressed in the form of mean ± standard deviation (*n* = 3). Data of the two groups were compared using independent samples *t*-test. One-way ANOVA (followed by Tukey posthoc test) was electively used for data comparison between multiple groups. Data were compared between groups at different time points using repeated measures ANOVA and Tukey posthoc test.

To know more about the role of miR-342-3p in M2-EVs, miR-342-3p expression was interfered in M2 TAMs. The results showed that the miR-342-3p level of M2-EVs in anti-miR-342-3p group was significantly lower than that in anti-NC group (t = 11.244, *p* < 0.001) (Fig. 3A). Subsequently, M2-EV-anti miR-342-3p was co-cultured with RCC cells, and miR-342-3p expression in RCC cells was tested to show a significant difference when compared with the control group (ACHN: F = 29.018, *p* < 0.001; 769-P: F = 37.155, *p* < 0.001). Compared with M2-EV-anti NC group, the expression of miR-342-3p in M2 EVs anti miR-342-3p group decreased more significantly (ACHN: q = 7.864, *p* = 0.004; 769-P: q = 11.350, *p* = 0.001) (Fig. 3B). Further biological experiments showed significantly enhanced cell viability (ACHN: F = 16.220, *p* < 0.001; 769-P: F = 19.721, *p* < 0.001. Fig. 3C), invasion (ACHN: F = 64.249, *p* < 0.001; 769-P: F = 75.923, *p* < 0.001. Fig. 3D, Fig. S2C) and migration (ACHN: F = 73.947, *p* < 0.001; 769-P: F = 56.465, *p* < 0.001. Fig. 3E, Fig. S2D) in the presence of M2-EVs-anti NC as compared to the control, while reverse trends were observed in M2-EVs-anti miR-342-3p group *vs*. M2-EVs-anti NC group (all *p* < 0.001) (Figs. 3C–3E). In the further Western blot assay for epithelial-mesenchymal transition (EMT)-related proteins in RCC, there was a significant decline in E-cadherin (ACHN: q = 16.459, *p* < 0.001; 769-P: q = 14.352, *p* < 0.001) while an evident elevation in Vimentin (ACHN: q = 17.045, *p* < 0.001; 769-P: q = 13.716, *p* < 0.001) in M2-EVs-anti NC group *vs*. control. To the contrary, opposite expressing trend was noted in M2-EVs-anti miR-342-3p group *vs*. M2-EVs-anti NC group (E-cadherin, ACHN: q = 14.846, *p* < 0.001; 769-P: q = 13.360, *p* < 0.001; Vimentin, q = 15.534, *p* < 0.001; 769-P: q = 12.640, *p* < 0.001) (Fig. 3F, Fig. S3B). Altogether, M2-EVs derived miR-342-3p can promote RCC cell viability and motility.

### M2-EVs-miR-342-3p prevents CEP55 degradation by targeted inhibition of NEDD4L in RCC cells

**Figure 4:**
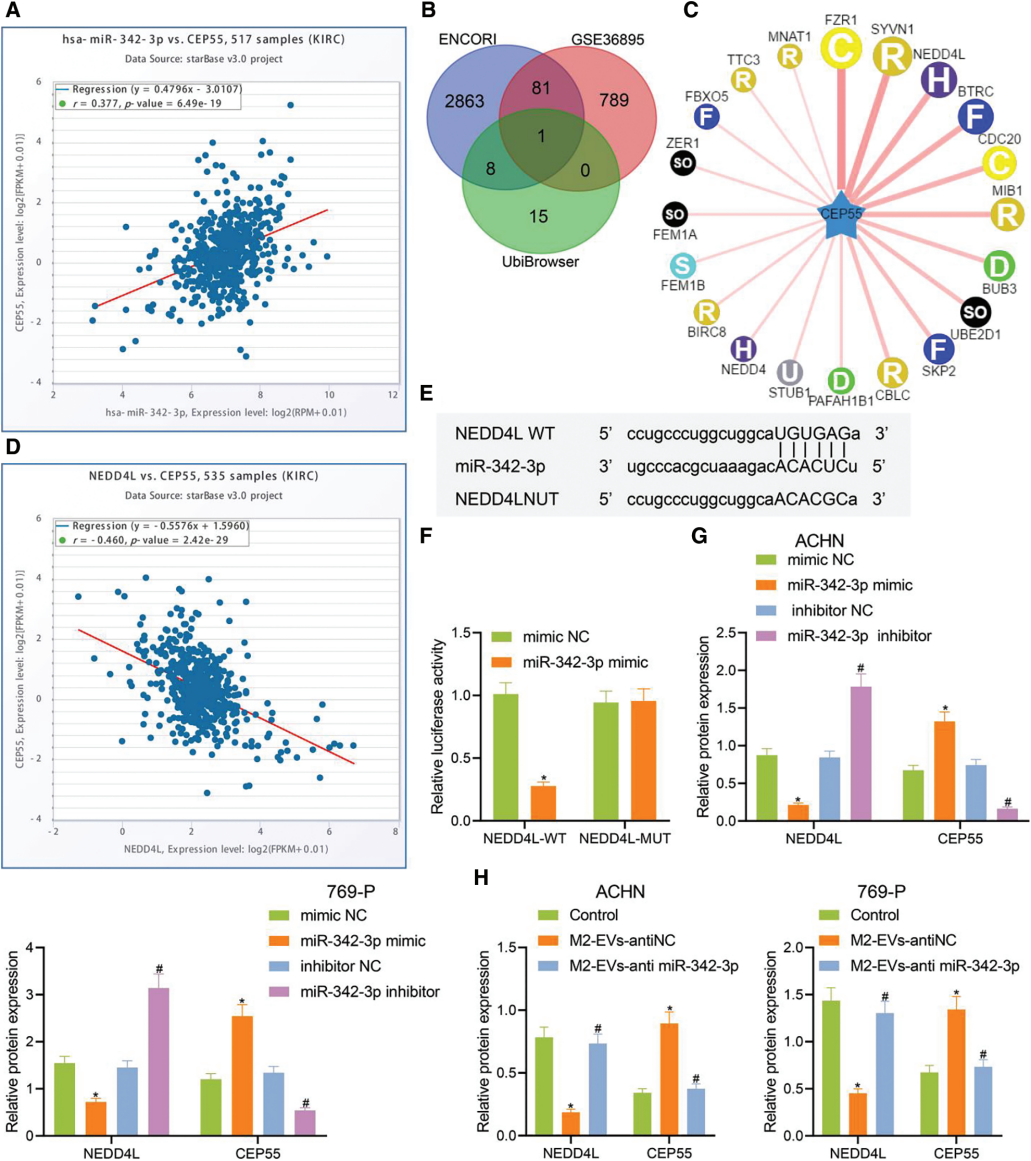
miR-342-3p modulates CEP55 expression by suppressing NEDD4L. (A) ENCORI database retrieval showing the correlation between miR-342-3p and CEP55 in RCC; (B) Venn diagram showing the overlapped genes from putative targets for miR-342-3p on ENCORI, differentially down-regulated genes in GSE36895 and E3 ubiquitin ligase for CEP55 on UbiBrowser; (C) Predicted network diagram of E3 ubiquitin ligases of modulating CEP55 on UbiBrowser; (D) Correlation between miR-342-3p and CEP55 in RCC on ENCORI; (E) Putative miR-342-3p binding sites on NEDD4L on Starbase (http://starbase.sysu.edu.cn/) website; (F) Dual luciferase reporter gene assay for HEK293 cells co-transfected by NEDD4L-WT or NEDD4L-MUT with mimic NC or miR-342-3p minic plasmids; (G) Protein levels of NEDD4L and CEP55 in RCC cells treated with mimic NC, miR-342-3p mimic, inhibitor NC or miR-342-3p inhibitor by Western blot; (H) Protein levels of NEDD4L and CEP55 in RCC cells treated with M2-EVs-anti NC or M2-EVs-anti miR-342-3p by Western blot. * indicates *p* < 0.05, compared to the mimic NC group or control group; # represents *p* < 0.05, compared to inhibitor NC group or M2-EVs-anti NC group. Measurement data were expressed in the form of mean ± standard deviation (*n* = 3). Data of the two groups were compared using independent samples *t*-test. One-way ANOVA (followed by Tukey posthoc test) was electively used for data comparison between multiple groups.

As previously documented, CEP55 showed abundant expression and played a tumor-promoting role in RCC [5]. Chen et al. also found that CEP55 potentiated EMT of RCC cells via activating PI3K/AKT/mTOR pathway [25]. Additionally, the ENCORI data implied a positive correlation of miR-342-3p with CEP55 in RCC (Fig. 4A).

To further investigate whether CEP55 was involved in M2-EVs-miR-342-3p dependent regulation in the tumorigenesis and development of RCC, potential downstream target genes for miR-342-3p were obtained on ENCORI and were then intersected with the differentially down-regulated genes in GSE36895 dataset and the E3 ubiquitin ligase of CEP55 predicted by the UbiBrowser database (Figs. 4B–4C). In the meantime, ENCORI data revealed a negative correlation between NEDD4L and CEP55 in RCC (Fig. 4D). Therefore, NEDD4L was eventually selected as the target gene.

A retrieval on Starbase showed potential miR-342-3p binding sites on NEDD4L 3′UTR (Fig. 4E). The dual-luciferase assay indicated a decline in luciferase signal in NEDD4L-WT group *vs*. mimic NC group (t = 13.273, *p* < 0.001), yet the signal varied not statistically in NEDD4L-MUT group *vs*. mimic NC group (t = 0.170, *p* = 0.873) (Fig. 4F). Thus, miR-342-3p can specifically inhibit the NEDD4L gene.

The associations among miR-342-3p, NEDD4L and CEP55 were further investigated. We then transfected RCC cells with miR-342-3pmimic/inhibitor. As detected by Western blot, no expression difference was observed in mimic-NC group *vs*. inhibitor-NC group (all *p* > 0.001). In miR-342-3p mimic group *vs*. mimic-NC group, miR-342-3p and CEP55 expressions were remarkably increased, and NEDD4L expression was observably decreased (all *p* < 0.001). In miR-342-3p inhibitor group *vs*. inhibitor-NC group, miR-342-3p, CEP55 and NEDD4L showed the reverse expression trend (all *p* < 0.001, Annex I) (Fig. 4G, Fig. S3C). Furthermore, RCC cells were treated with M2-EVs. It was observed that NEDD4L expression was significantly decreased (ACHM: F = 76.823, *p* < 0.001; 769-P: F = 70.154, *p* < 0.001) while CEP55 expression was increased (ACHM: F = 78.031, *p* < 0.001; 769-P: F = 41.620, *p* < 0.001) compared with the control group. To the contrary, NEDD4L (ACHM: q = 14.487, *p* < 0.001; 769-P: q = 13.379, *p* < 0.001) showed elevated expression and CEP55 (ACHM: q = 14.842, *p* < 0.001; 769-P: q = 10.641, *p* < 0.001) displayed reduced expression in M2-EVs-anti miR-342-3p group *vs*. M2-EVs-anti NC group (Fig. 4H, Fig. S3D). Collectively, the data indicate that M2-EVs-miR-342-3p can prevent CEP55 degradation by suppressing NEDD4L in RCC cells.

### M2-EVs-miR-342-3p/NEDD4L/CEP55 axis enhances RCC growth and metastasis

**Figure 5:**
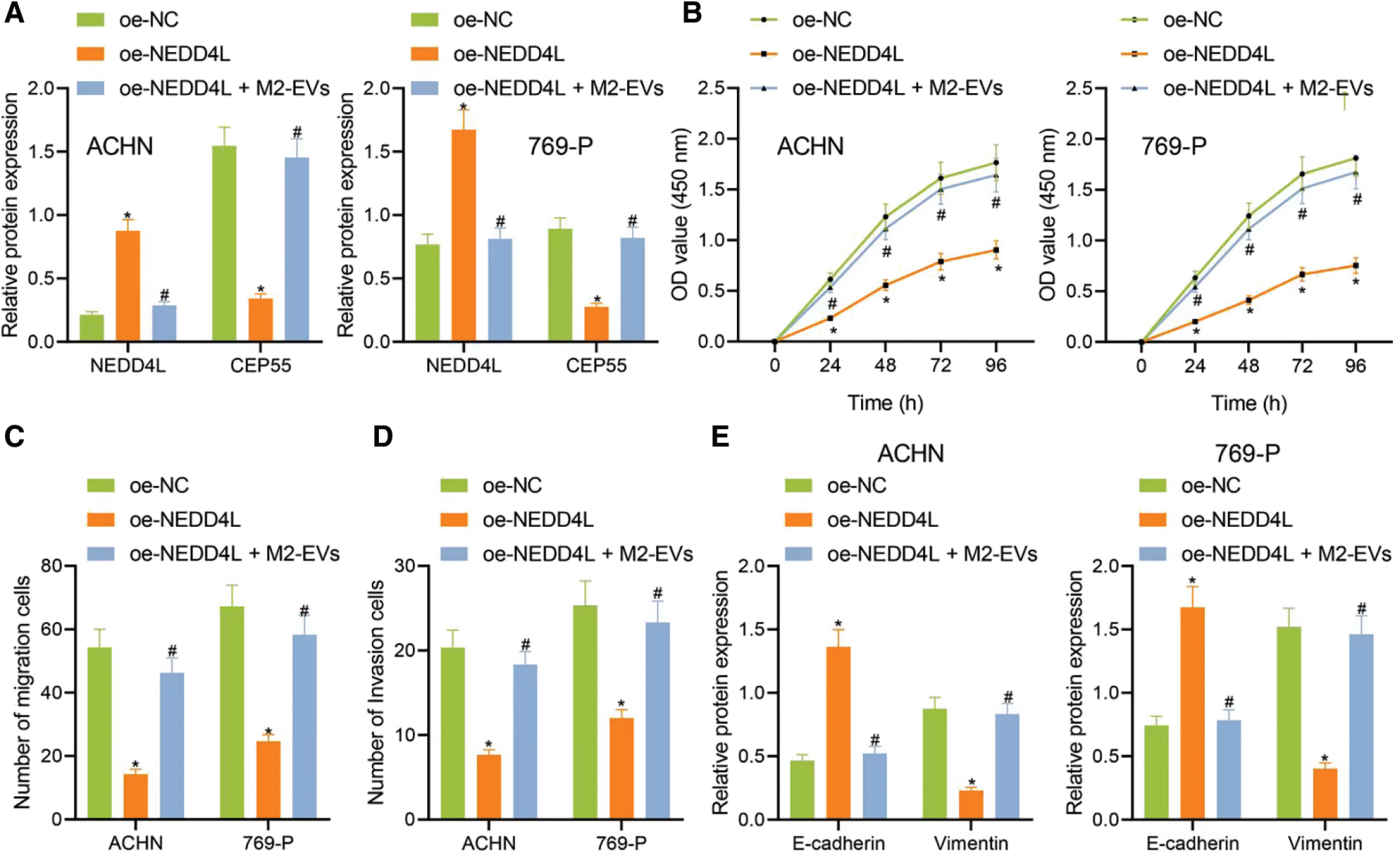
M2-EVs-miR-342-3p functions on proliferation, migration and invasion of RCC cells via regulating NEDD4L/CEP55 axis. (A) Western blot analysis for NEDD4L and CEP55 in RCC cells treated with oe-NEDD4L or oe-NEDD4L+M2-EVs; (B) RCC cell viability for each treatment group by CCK-8 assay; (C, D) RCC cell migration and invasion abilities for each treatment group by Transwell assay; (E) Western blot analysis for EMT markers E-cadherin and Vimentin expression in each treatment group of RCC cells. * indicates *p* < 0.05, compared to the oe-NC group; # represents *p* < 0.05, compared to the oe-NEDD4L group. Measurement data were expressed in the form of mean ± standard deviation (*n* = 3). Data of the two groups were compared using independent samples *t*-test. One-way ANOVA (followed by Tukey posthoc test) was electively used for data comparison between multiple groups. Data were compared between groups at different time points using repeated measures ANOVA and Tukey posthoc test.

To further investigate the involvement of NEDD4L/CEP55 axis in miR-342-3p-regulated RCC, we transfected RCC cells with pcDNA-3.1-NEDD4L (oe-NEDD4L) and extracted M2-EVs to treat RCC cells. As reflected by Western blot, NEDD4L expression (ACHN: q = 20.427, *p* < 0.001; 769-P: q = 13.999, *p* < 0.001) was increased and CEP55 expression (ACHN: q = 17.728, *p* < 0.001; 769-P: q = 15.175, *p* < 0.001) was significantly decreased in oe-NEDD4L group *vs*. oe-NC group. Meanwhile, NEDD4L (ACHN: q = 18.172, *p* < 0.001; 769-P: q = 13.294, *p* < 0.001) was starkly downregulated and CEP55 (ACHN: q = 15.814, *p* < 0.001; 769-P: q = 13.404, *p* < 0.001) was sharply up-regulated in oe-NEDD4L+M2-EVs group *vs*. oe-NEDD4L group (Fig. 5A, Fig. S3E).

Subsequently, biological functional experiments were devised to uncover significantly weakened cell viability (all *p* < 0.001) (Fig. 5B), migration (ACHN: q = 16.036, *p* < 0.001; 769-P: q = 13.803, *p* < 0.001) (Fig. 5C, Fig. S2E) and invasion (ACHN: q = 14.363, *p* < 0.001; 769-P: q = 10.106, *p* < 0.001) (Fig. 5D, Fig. S2F) in oe-NEDD4L group *vs*. oe-NC group, while profoundly enhanced abilities of RCC cells after M2-EVs application when compared with oe-NEDD4L alone (all *p* < 0.001) (Figs. 5B–5D, Figs. S2E, S2F). Additionally, NEDD4L overexpression led to increased E-cadherin while decreased Vimentin, which could be restored after M2-EVs treatment (all *p* < 0.001) (Fig. 5E, Fig. S3F).

**Figure 6:**
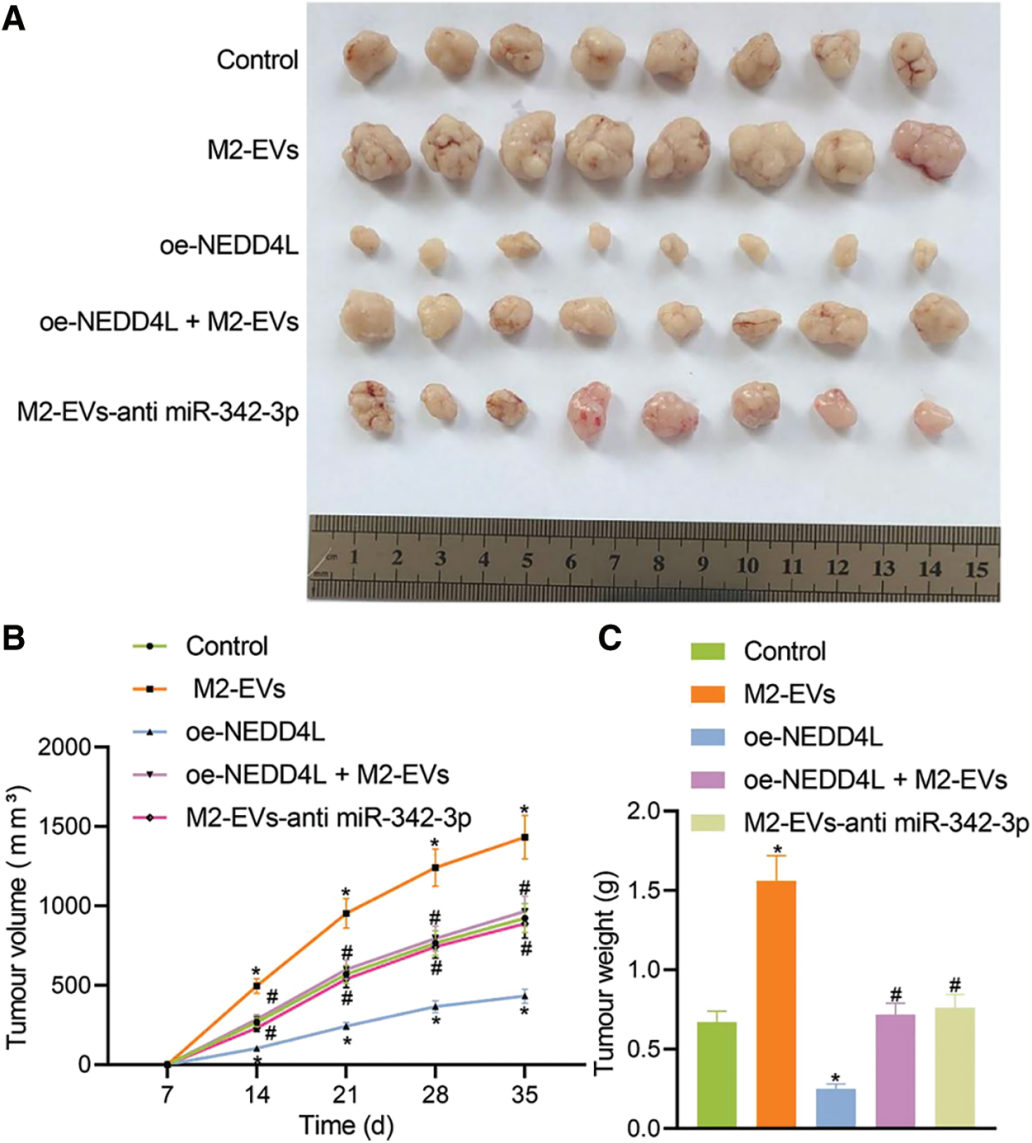
M2-EVs-miR-342-3p potentiates tumorigenesis of RCC via the NEDD4L/CEP55 axis *in vivo*. (A) Representative images of xenograft tumour in M2-EVs, oe-NEDD4L, oe-NEDD4L+M2-EVs, and M2-EVs-anti miR-342-3p treated nude mice; (B) Changes of tumor volumes in each treatment group in xenograft tumor model; (C) Tumor weight in each treatment group in xenograft tumor model. N = 8 for each group. * indicates *p* < 0.05 compared to the Control group, and # indicates *p* < 0.05 compared to the M2-EVs group. Measurement data were expressed in the form of mean ± standard deviation (*n* = 3). One-way ANOVA (followed by Tukey posthoc test) was electively used for data comparison between multiple groups. Data were compared between groups at different time points using repeated measures ANOVA and Tukey posthoc test.

*In vivo* animal experiment was then conducted. Empty RCC cells or cells overexpressing NEDD4L were respectively subcutaneously injected into the nude mice, along with the tail injection of M2-EVs. It was found that NEDD4L overexpression resulted in reduced tumor growth speed and weight, while the presence of M2-EVs exerted a tumor-promoting effect. Additionally, the tumor growth speed and weight tended to be significantly reduced in oe-NEDD4L+M2-EVs group and M2-EVs-anti miR-342-3p group *vs*. M2-EVs group (all *p* < 0.001) (Figs. 6A–6C).

**Figure 7:**
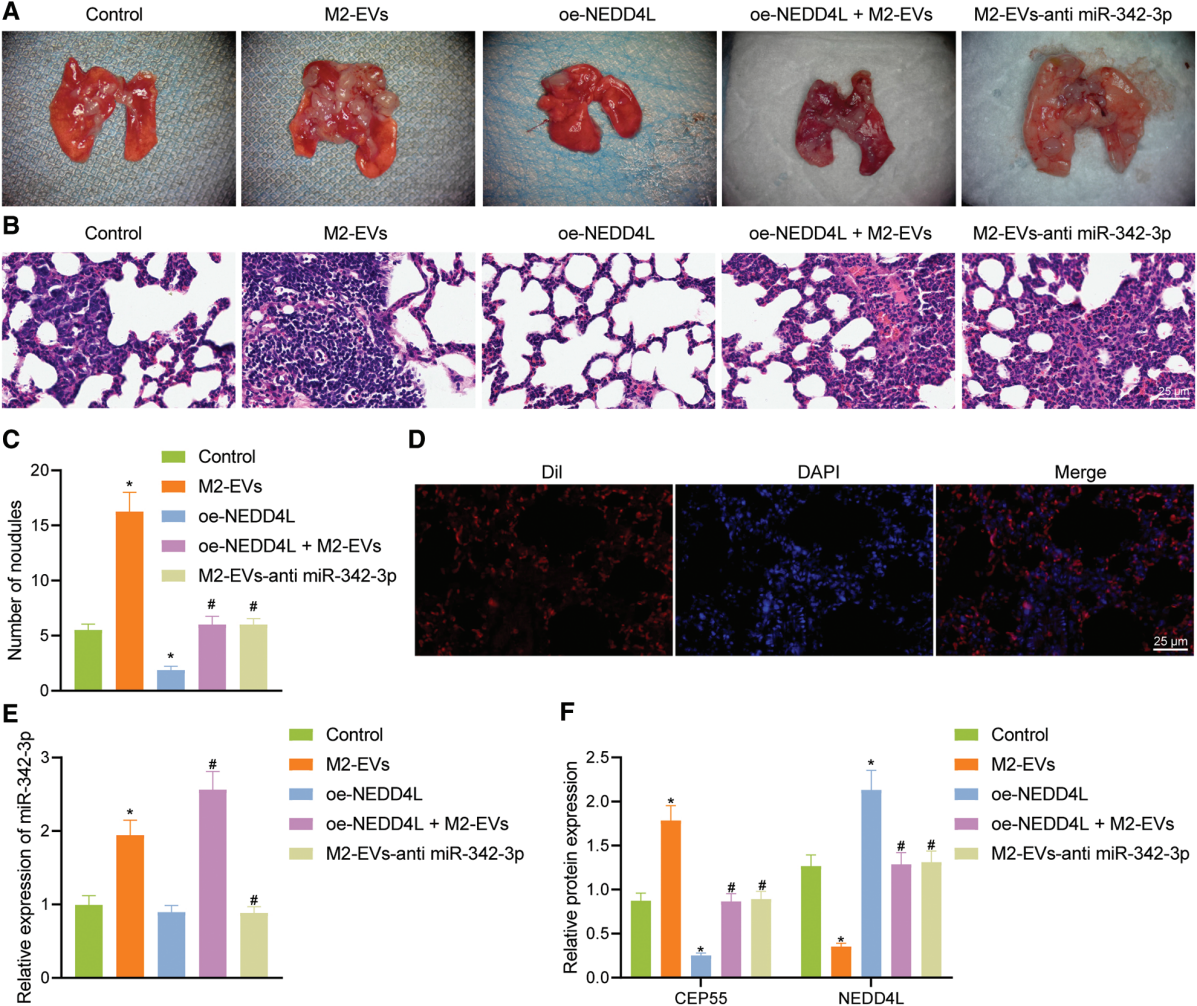
M2-EVs-miR-342-3p potentiates metastasis of RCC via the NEDD4L/CEP55 axis *in vivo*. (A) Representative images of lung metastasis noudules in M2-EVs, oe-NEDD4L, oe-NEDD4L+M2-EVs, and M2-EVs-anti miR-342-3p treated nude mice; (B) H&E staining on lung tissues in each treatment group in nude mouse model of lung metastasis (scale bar = 25 μm); (C) Lung metastasis noudules in each treatment group in nude mouse model of lung metastasis; (D) Fluorescence images of DiI-labeled EVs (Red) in lung tissue (scale bar=25 μm); (E) RT-qPCR assay for miR-342-3p expression in lung tissue in each treatment group in nude mouse model of lung metastasis; (F) Western blot analysis for NEDD4L and CEP55 protein expression in lung tissue in each treatment group in nude mouse model of lung metastasis. * indicates *p* < 0.05 compared to the Control group, and # indicates *p* < 0.05 compared to the M2-EVs group. Measurement data were expressed in the form of mean ± standard deviation (*n* = 3). One-way ANOVA (followed by Tukey posthoc test) was electively used for data comparison between multiple groups. Data were compared between groups at different time points using repeated measures ANOVA and Tukey posthoc test.

In lung metastasis models, lung metastasis was much frequent in control (q = 11.006, *p* < 0.001) and M2-EVs (q = 43.644, *p* < 0.001) groups *vs*. oe-NEDD4L group. In the meantime, the metastatic potential in M2-EVs group was also much higher as compared to oe-NEDD4L+M2-EVs group (q = 31.120, *p* < 0.001) and M2-EVs-anti miR-342-3p group (q = 31.120, *p* < 0.001) (Figs. 7A–7C). DiI-labeled EVs could be observed in the metastatic lung tissue (Fig. 7D). Furthermore, miR-342-3p, NEDD4L and CEP55 levels were measured in lung tissue of each group. As analyzed, relative to control group, in M2-EVs group, miR-342-3p (q = 16.455, *p* < 0.001) (Fig. 7E) and CEP55 (q = 20.732, *p* < 0.001) (Fig. 7F, Fig. S3G) levels were elevated and NEDD4L (q = 30.413, *p* < 0.001) (Fig. 7F) was reduced. NEDD4L (q = 16.870, *p* < 0.001) (Fig. 7F) showed higher expression while CEP55 (q = 27.814, *p* < 0.001) (Fig. 7F) was lower expressed in oe-NEDD4L *vs*. control. In comparison to M2-EVs, the increase of miR-342-3p (q = 10.761, *p* < 0.001) (Fig. 7E) and CEP55 (q = 17.514, *p* < 0.001) (Fig. 7F) levels in oe-NEDD4L+M2-EVs group was much more significant. Comparing M2-EVs group to M2-EVs-anti miR-342-3p group, a profound increase in miR-342-3p (q = 18.352, *p* < 0.001) (Fig. 7E) and CEP55 (q = 16.222, *p* < 0.001) (Fig. 7F) expressions and a reduction in NEDD4L (q = 30.887, *p* < 0.001) (Fig. 7F) were noted (all *p* < 0.05). Taken together, M2-EVs-miR-342-3p promotes growth and metastasis of RCC via the NEDD4L/CEP55 axis.

### M2-EVs-miR-342-3p mediates E3 ubiquitin ligase NEDD4L to affect CEP55 degradation

Existing research demonstrated that NEDD4L as an E3 ubiquitin ligase could promote the degradation of related proteins [26]. Besides, CEP55 was reported to promote the EMT of RCC cells via activating PI3K/AKT/mTOR pathway [25]. It was thus speculated that M2-EVs-miR-342-3p participated in RCC occurrence and progression probably via the NEDD4L/CEP55/PI3K/AKT/mTOR axis.

**Figure 8:**
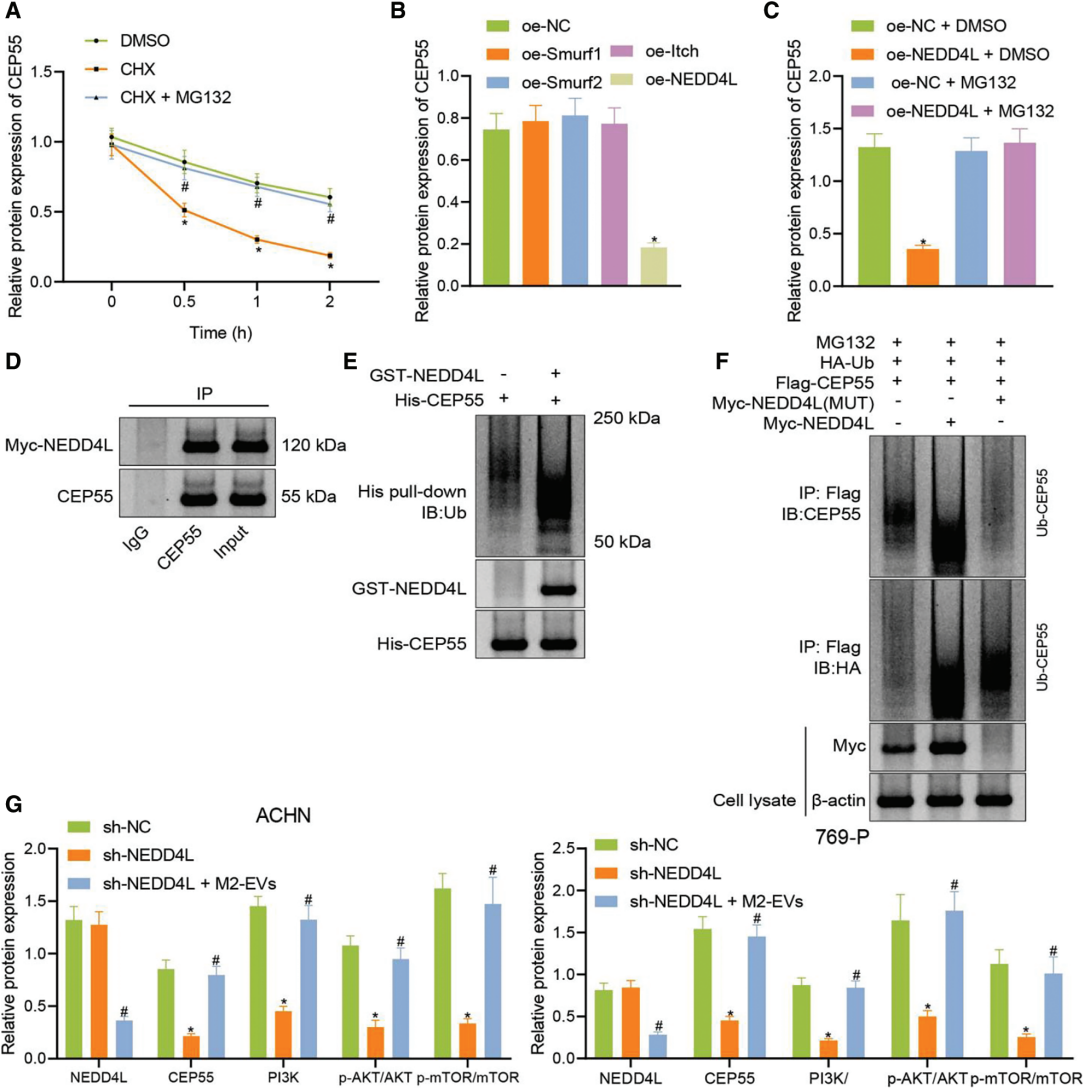
M2-EVs regulates E3 ubiquitin ligase NEDD4L to affect CEP55 ubiquitination and degration and active PI3K/AKT/mTOR signaling pathway. (A) Changes of CEP55 level in ACHN cells treated with CHX or CHX-MG132; (B) Western blot analysis for CEP55 expression after overexpressing ubiquitin ligases Smurf1, Smurf2, Itch and NEDD4L; (C) Western blot analysis for CEP55 expression after overexpressing NEDD4L with or without treatment of MG132; (D) Co-IP assay to verify the interaction between NEDD4L and CEP55 in ACHN cells (IgG as negative control and Input as positive control); (E) *In vitro* GST-pull down assay to verify the interaction between GST-NEDD4L and His-CEP55 in ACHN cells (GST as negative control and Input as positive control); (F) Ubiquitination of CEP55 by NEDD4L as detected by IP with anti-Flag antibody followed by immunoblotting with anti-CEP55 or anti-HA antibody; (G) Protein levels of NEDD4L, CEP55, PI3K/AKT/mTOR signaling pathway markers (PI3K, p-AKT-Ser473 and p-mTOR-ser2448) in ACHN cells after treatment with sh-CEP55 or sh-CEP55+M2-EVs. * indicates *p* < 0.05, compared to the DMSO, oe-NC, oe-NC+DMSO or sh-NC groups; # indicates *p* < 0.05, compared to oe-NC+MG132 or sh-CEP55 groups. Measurement data were expressed in the form of mean ± standard deviation (*n* = 3). One-way ANOVA (followed by Tukey posthoc test) was electively used for data comparison between multiple groups. Data were compared between groups at different time points using repeated measures ANOVA and Tukey posthoc test.

To assess whether CEP55 could be ubiquitinated and degraded by the E3 ubiquitin ligase NEDD4L in RCC, CEP55 protein expression was measured in RCC cells. It was revealed that CEP55 protein decreased with prolonged time in the presence of CHX, as compared to dimethyl sulfoxide (DMSO) group (all *p* < 0.001), while the decrease slowed after CHX-MG132 treatment (all *p* < 0.001) (Fig. 8A). Following overexpression treatment for ubiquitin ligases Smurf1, Smurf2, Itch and NEDD4L, a decline of CEP55 was only observed upon NEDD4L overexpression (all *p* < 0.001), oe-NEDD4L group compared with other groups (Fig. 8B, Fig. S3H), which was suppressed after MG132 intervention (q = 15.469, *p* < 0.001; oe-NEDD4L+DMSO *vs*. oe-NEDD4L+MG132) (Fig. 8C, Fig. S3I).

Co-IP and GST pull-down assays were performed in the presence of MG132 to test the relationship between NEDD4L and CEP55 at the protein level. In the meantime, endogenous CEP55 was detected after expression of Myc-labeled NEDD4L in cells. As displayed in (Fig. 8D), Myc-NEDD4L could be co-precipitated with anti-CEP55 antibody but not with control immunoglobulin IgG. It reveals the interaction of NEDD4L with endogenous CEP55 in RCC cells.

To validate the direct interaction between the two, a GST-pull-down assay was conducted with *in vitro* synthetic His-CEP55 and GST-NEDD4L (Fig. 8E). The effect of NEDD4L overexpression on CEP55 ubiquitination was then explored. As shown in (Fig. 8F), NEDD4L could promote the ubiquitination and degradation of CEP55, while such effect was reduced when mutations occurred in NEDD4L. This implies that CEP55 ubiquitination and degradation can be modulated by E3 ubiquitin ligase NEDD4L.

Finally, to gain more insight into the miR-342-3p/NEDD4L/CEP55/PI3K/AKT/mTOR axis, CEP55 was silenced in RCC cells, followed by co-culture with M2-EVs. In the results, CEP55 expression (ACHN: q = 16.059, *p* < 0.001; 769-P: q = 15.900, *p* < 0.001) was silenced after short hairpin(sh)-CEP55 transfection, along with reduction in PI3K (ACHN: q = 15.259, *p* < 0.001; 769-P: q = 1476, *p* < 0.001) expression and decrease in phosphorylation levels of AKT-Ser473 (ACHN: q = 15.007, *p* < 0.001; 769-P: q = 8.861, *p* < 0.001) and mTOR-ser2448 (ACHN: q = 13.166, *p* < 0.001; 769-P: q = 9.915, *p* < 0.001), yet NEDD4L expression was not significantly changed (ACHN: q = 0.746, *p* = 0.861; 769-P: q = 0.792, *p* = 0.846). Nevertheless, compared with sh-CEP55, co-culture with sh-CEP55 and M2-EVs decreased NEDD4L (ACHN: q = 15.010, *p* < 0.001; 769-P: q = 13.879, *p* < 0.001) protein expression and posed promotive effects on CEP55 (ACHN: q = 14.662, *p* < 0.001; 769-P: q = 14.582, *p* < 0.001) and PI3K (ACHN: q = 13.293, *p* < 0.001; 769-P: q = 15.676, *p* < 0.001) protein levels, as well as phosphorylation levels of AKT-S473 (ACHN: q = 12.536, *p* < 0.001; 769-P: q = 9.761, *p* < 0.001) and mTOR-ser2448 (ACHN: q = 11.645, *p* < 0.001; 769-P: q = 8.601, *p* < 0.001) were increased (Fig. 8G, Fig. S3J).

Altogether indicates that M2-EVs inhibit the E3 ubiquitin ligase NEDD4L from preventing the ubiquitination and degradation of CEP55 and activating the PI3K/AKT/mTOR signaling pathway.

## Discussion

It is well established that the formation and maintenance of a cancer niche profoundly depend on its surrounding microenvironment, which consists of a network of reciprocal cell types such as endothelial cells, inflammatory cells, fibroblasts, stem cells, and immune cells [12,27]. TAMs are the most prominent tumor-infiltrating immune cells within the TME, and despite growing research, the regulatory crosstalk between TAM and RCC cells, especially how macrophages modulate various hallmarks of RCC during tumor progression remains not completely understood. Here, we found that elevated exogenous miR-342-3p within M2-EVs effectively disrupted NEDD4L expression and thus prevented the ubiquitination and degradation of CEP55. The up-regulated CEP55 subsequently activated the PI3K/AKT/mTOR pathway and strongly induced the proliferative, migratory and invasive properties of RCC cells.

Dysregulated miRNAs play a critical role in carcinogenesis [28]. miR-342-3p, localized to 14q32, has become as an important cancer-related miRNA in human cancers [29]. This miRNA is frequently down-regulated in hepatocellular carcinoma, non-small cell lung cancer, and gallbladder cancer, and acts as a tumor suppressor [22,30,31]. However, its functional significance in RCC is still poorly elucidated. In this study, we intriguingly verified a significant up-regulated expression of miR-342-3p in RCC tissues compared with normal controls, revealing that miR-342-3p was a carcinogenic factor rather than a suppressor implicated in the development and progression of RCC. In addition, we uncovered the highly expressed miR-342-3p in M2 macrophage-derived EVs. Accumulating evidence has supported the important roles of EV-derived miRNAs in RCC [20–21,32]. For instance, serum exosomal miR-210 originating from tumor tissue has been regarded as a novel diagnostic marker and prognostic predictor for ccRCC progression [21]. Additionally, urinary exosomal miR-30c-5p targets heat-shock protein 5 and inhibits ccRCC progression, which has considerable potential as a diagnostic biomarker for early-stage ccRCC [20]. Moreover, EVs shuttled miR-31-5p can transfer resistance information and promote sorafenib resistance in RCC by directly targeting MutL homolog 1, thereby both miR-31-5p and its target gene are likely to be predictive biomarkers and therapeutic targets for sorafenib resistance [32]. In current work, we demonstrated that M2-EVs could carry and transmit miR-342-3p into RCC cells, thereafter driving RCC proliferative, migratory and invasive capacities *in vitro* and *in vivo*. This observation indicated that miR-342-3p appeared to be a novel and effective therapeutic target gene for RCC.

Another important observation in this study was that miR-342-3p could bind to NEDD4L and down-regulate its expression. NEDD4L is an E3 ubiquitin ligase that regulates channel internalization and turnover [33]. It appears to possess roles in multiple cell processes, such as transporter modulation, autophagy and signal transduction [33]. NEDD4L level is significantly changed, and it exhibits distinct functions in different carcinomas by regulating certain major pathways (such as TGF-β, WNT and EGFR signaling pathways) [34,35]. In gallbladder cancer, NEDD4L is significantly up-regulated and exerted a pro-oncogenic role through regulation of matrix metallopeptidase 1 and 13 genes transcription [36], whereas in prostate cancer, down-regulation or loss of function of NEDD4L (that can exert an antitumor activity via regulating the TGFβ1 signaling) is observed and proposed to be associated with the malignancy [37]. Also, decreased NEDD4L expression in NSCLC is found to be more tumor aggressive and can predict poorer survival time [34,38]. Here, we showed that NEDD4L expression was decreased in RCC cell lines by miR-342-3p delivered in M2-EVs, thus consequently promoting the RCC cell migration and invasion. Our observation was inconsistent with other reports of the low expression of NEDD4L in ccRCC [37,39]. All these elucidated the tumor suppressive role of NEDD4 in RCC carcinogenesis. Meanwhile, we found that transfer of M2-EVs-drived miR-342-3p into RCC cells to target NEDD4L diminished the degradation of CEP55. NEDD4L has been widely reported to modulate multiple signaling pathways in tumor cells through the ubiquitination and degradation pathways [40,41]. For example, ERBB3 (HER3), one of the EGFR family proteins, can undergo NEDD4L-mediated ubiquitination and degradation to down-regulate the signal transduction pathways of occurrence and development in cancers [42]. Additionally, NEDD4L is involved in ULK1 ubiquitination and degradation, thereby playing vital roles in initiating autophagy and maintaining redox homeostasis [43,44]. Our study verified the negative regulatory relationship between NEDD4L and CEP55; thus, the above reports would support our hypothesis that NEDD4L exerts the ubiquitination regulation effect on CEP55. CEP55, a centrosome- and midbody-associated protein, is critical for cell cycle progression and cytokinesis [45]. Many studies have revealed that CEP55 serves a pivotal role in the cell cycle and survival through the regulation of the PI3K/AKT pathway [25,45,46]. For instance, Li et al. has shown that CEP55 can promote proliferation and inhibit apoptosis via the PI3K/AKT/p21 signal pathways, further attributing to the carcinogenesis and progression of glioma cells [46]. Additionally, Chen et al. have demonstrated that the PI3K/AKT/mTOR pathway is capable of regulating the effects of CEP55 on the migration, invasion and EMT of RCC cells and might be used as an effective prognostic marker [25]. In consistent with this, our study also verified that up-regulated CEP55 could sustain RCC growth, proliferation and metabolism through activation of the PI3K/AKT/mTOR pathway. In conjunction with existing evidence, we preliminarily indicated that M2-EVs encapsulated miR-342-3p could promote the CEP55 expression by targeting NEDD4L and inhibiting NEDD4L expression, thus consequently promoting the proliferative, migratory and invading properties of RCC cells via activation of the PI3K/AKT/mTOR pathway.

In fact, PI3K/AKT pathway is modestly mutated but highly activated in RCC [47]. Recent studies have evidenced that classical activation of the PI3K/AKT network is not always triggered by extracellular stimuli and transmembrane protein receptors [47]. Currently, miRNAs are emerging as a new class of important regulators of the PI3K/AKT pathway. For example, miR-122 is proved to be a positive modulator of the PI3K/AKT signaling, that can promote proliferation, invasion, and migration of RCC cells [48]. Additionally, miR-182-5p is in a down-regulated expression pattern of AKT, and its reduction results in AKT activation and subsequent RCC proliferation [49]. These results partially supported that miR-342-3p could be identified to be a novel regulator that contributed to aberrant activation of the PI3K/AKT pathway in RCC, representing a promising target for RCC therapy. Moreover, a previous study has reported that in the presence of tumors, EV-enrichment may represent an epigenetic silencing mechanism whereby ccRCC maintains tumor development and growth by activating the PI3K/AKT pathway [32]. The overall activation of the PI3K/AKT pathway in ccRCC is higher than in other cancers, suggesting that dysregulation of the PI3K/AKT pathway in RCC may be a consequence of EV-mediated epigenetic mechanisms [47,50].

Altogether, findings obtained in our study concluded that M2-EVs-drived miR-342-3p could play an important role in the invasion and migration process of RCC, and had the potential to be used as a therapeutic biomarker. However, we recognize that additional mechanisms may be involved in such pathways, which warrants further exploration.

## Conclusion

**Figure 9:**
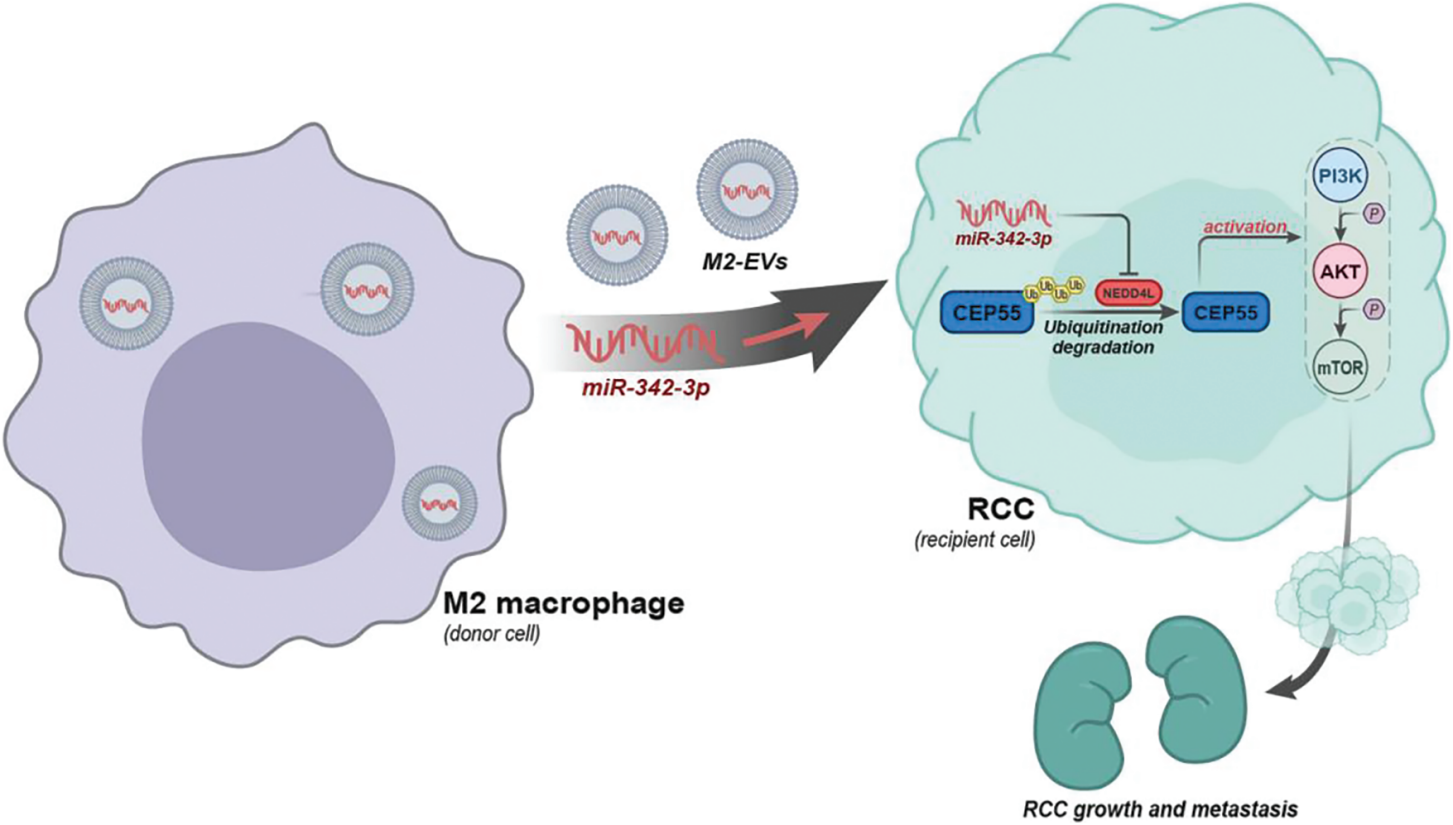
Schematic diagram of the molecular mechanism by which M2-EVs participate in RCC tumorigenesis and progression by delivering miR-342-3p and mediating the NEDD4L/CEP55 axis.

To sum up, a preliminary conclusion can be obtained in this research that M2 macrophages may carry miR-342-3p through EVs to target NEDD4L in RCC cells, inhibit the ubiquitination degradation of CEP55 and activate the PI3K/AKT/mTOR signaling pathway, thereby promoting the growth and metastasis of RCC (Fig. 9). This finding provides a novel therapeutic target in future RCC treatment.

## Data Availability

The datasets used and/or analysed during the current study are available from the corresponding author on reasonable request.

## List of Abbreviations

CEP55: centrosomal protein 55; Co-IP: co-immunoprecipitation; EMT: epithelial-mesenchymal transition; EVs: extracellular vesicles; FBS: fetal bovine serum; HUVECs: human umbilical vein endothelial cells; NEDD4L: neural precursor cell expressed developmentally down-regulated gene 4-like; NHC, National health commission; RCC: renal cell carcinoma; TAMs: tumor-associated macrophages; TEM: transmission electron microscope; TME: tumor microenvironment.
